# Effects of Integrated Community-Based Care and Group Microfinance on Antiretroviral Therapy Adherence Among Adults Living With HIV in Western Kenya

**DOI:** 10.1177/23259582251414566

**Published:** 2026-02-19

**Authors:** Emily T. O’Neill, Juddy Wachira, Joshua Juma, Ben Mosong, Catherine Kafu, Marta Wilson-Barthes, Sonak D. Pastakia, Dan N. Tran, Becky L. Genberg, Omar Galárraga

**Affiliations:** 1 Department of Health Services Policy and Practice, Brown University School of Public Health, Providence, RI, USA; 2 130188 Academic Model Providing Access to Healthcare (AMPATH), Eldoret, Kenya; 3 Behavioral Sciences, Moi University College of Health Sciences, School of Medicine, Eldoret, Kenya; 4 Center for Global Public Health, 174610 Brown University School of Public Health, Providence, RI, USA; 5 Department of Pharmacy Practice, Purdue University College of Pharmacy, Indianapolis, IN, USA; 6 Department of Pharmacy Practice, School of Pharmacy, Temple University, Philadelphia, PA, USA; 7 Department of Epidemiology, Johns Hopkins University Bloomberg School of Public Health, Baltimore, MD, USA

**Keywords:** ART adherence, access to medication, community-based care, group microfinance, western Kenya

## Abstract

**Background:**

Despite the introduction of single-pill antiretroviral therapy (ART), adherence remains suboptimal in Sub-Saharan Africa. The Harambee study evaluated the effects of delivering integrated community-based (ICB) HIV care within small lending and savings groups called microfinance groups in western Kenya. Here, we explore the intervention's, a 2-arm cluster randomized trial, impact on ART adherence.

**Methods:**

We calculated the medication possession ratio (MPR) across 18 months at 3 time points using ART refill data from May 2021 to July 2023. As a secondary outcome, we assessed patient-reported 4-day ART adherence changes between study months 0 and 18. Outcomes were analyzed using linear regression models with treatment-by-time interaction terms to estimate time-varying treatment effects and month fixed effects, with standard errors clustered at the appropriate group level.

**Results:**

Baseline mean MPRs were 0.971 for microfinance group members receiving ICB care, 0.989 for microfinance groups receiving usual (facility-based) care, and 0.995 for frequency-matched usual care patients not engaged in microfinance. At 18 months, MPRs were significantly higher among microfinance groups receiving usual care (0.057, *P* < 0.001) and microfinance groups receiving ICB care (0.048, *P* < 0.001) compared to baseline. Four-day ART adherence ratios increased for participants enrolled in group microfinance with usual care (0.021, *P* = 0.05). Findings were consistent across all models and robustness checks.

**Conclusions:**

Combining ICB care with group microfinance significantly increased ART adherence and may contribute to increased HIV viral suppression.

## Plain Language Summary Title

Effects of Integrated Community-Based Care and Group Microfinance on HIV Treatment Adherence in Western Kenya

**Plain Language Summary:**

In Sub-Saharan Africa, many people living with HIV struggle to stay in care, even with better treatment options available. The Harambee study looked at how community-based HIV care linked with microfinance group participation (small loans and financial support) in western Kenya affected patients' ability to adhere to their HIV medication.

To measure this, researchers tracked how patients were picking up with their HIV medication refills from May 2021 to July 2023. They also interviewed patients about their medication adherence.

After 18 months, those who participated in both microfinance and facility-based care had a significant improvement in their HIV medication adherence. Additionally, involvement in microfinance groups increased how consistently patients reported their adherence.

Combining community-based care with microfinance not only improved adherence to HIV treatment but may also help in achieving better health outcomes for patients.

## Introduction

In 2017, Kenya became the second country in Sub-Saharan Africa (SSA) to introduce an optimized antiretroviral regimen containing dolutegravir, the drug of choice for many high-income countries for persons living with HIV (PLHIV).^1^ This single-pill combination antiretroviral therapy (ART) (dolutegravir, tenofovir, lamivudine) has a lower potential for resistance development, is easier to administer, and has fewer side effects than alternative ARTs, all of which can help achieve viral suppression more effectively.^2,3^

Successful HIV treatment depends not only on the availability of ART but also on sustained medication adherence. Adherence is defined as “the degree to which a person's behavior corresponds with the agreed recommendations from a health care provider.”^4^ In HIV care, adherence can be measured by the medication possession ratio (MPR). Near-perfect ART adherence, measured as an MPR ≥95%, is a key indicator of successful HIV viral suppression.^5,6^ However, recent clinical trials suggest that slightly lower levels of adherence may be sufficient for maintaining viral suppression.^7,8^ Nevertheless, these trials were conducted under highly controlled conditions with close monitoring and virologically suppressed patient populations, which may limit their generalizability. In real-world programmatic settings, optimal adherence remains a reliable strategy to sustain viral suppression and prevent onward transmission.^9^

Despite expanding access to better-tolerated ART, retention in HIV care remains suboptimal in SSA, with an estimated 52% of PLHIV achieving viral suppression.^10^ Decreased retention in HIV care can decrease ART adherence and subsequent viral suppression, increasing the likelihood of viral transmission and mortality.^11,12^ In western Kenya, primary barriers to retaining patients in HIV care are distance to facilities, inefficient vertical care delivery, and poor access to transportation and food.^11,13^ Negative societal beliefs about HIV, which can increase secrecy and minimize health-seeking behaviors or information sharing, can further decrease retention and subsequent ART _adherence.^14^ Efforts to increase retention in HIV health services ensure that patients can initially and continuously access ART and undergo clinical monitoring.^12^ Continuous and accessible clinical monitoring is needed for all PLHIV to observe ART adverse effects, assess for treatment failure, and promptly switch therapies to second or third-line agents as needed.^15^

Community-based care models have been adopted across SSA to decrease the strain on health systems while improving retention in care and decreasing HIV stigma.^16–18^ For patients in difficult-to-reach regions, community-based care can increase access to HIV services by decentralizing clinical care into the communities. Prior studies indicate that community-based HIV care is associated with increased viral suppression in both South Africa and Uganda, when compared to care provided at a health facility.^17^

The Harambee study in western Kenya tested the extent of integrated community-based (ICB) care delivered through group microfinance on HIV viral suppression and care retention.^19^ Here, microfinance refers to community-based savings and lending groups modelled after informal financial structures such as Rotating Savings and Credit Associations and Village Savings and Loan Associations, specifically the Group Integrated Savings for Health Empowerment (GISHE) groups implemented at Academic Model Providing Access to Healthcare (AMPATH).^20–24^ In this paper, we leverage the randomized trial design to estimate the effect of ICB care on ART adherence. We hypothesize that this model will improve adherence by addressing both clinical and social determinants of health, with the potential to generate economic benefits in conjunction with improved health outcomes.

## Methods

### Study Design

The Harambee study included a 2-arm cluster randomized trial from November 26^th^, 2020, to December sixth, 2023.^19^ The study was conducted within 2 counties in western Kenya, Busia and Trans Nzoia, by investigators affiliated with Academic Model Providing Access to Healthcare (AMPATH). Each county has a total of 24 rural health facilities (sub-county hospitals, health centers, and dispensaries) staffed by physicians, clinical officers, and nurses.^25^ Further details regarding the Harambee study, eligibility, and methodology have been published elsewhere.^19^ The reporting of this study conforms to the CONSORT 2025 statement (Supplemental Appendix 1).^26^

### Interventions

The primary intervention of the trial included integrated community-based (ICB) care for HIV, diabetes, and hypertension delivered by a clinical officer during regularly scheduled microfinance group meetings in the community. During care visits, a clinical officer delivered clinical consultations, diabetes and/or hypertension management, distribution of ART and noncommunicable disease medications, health education, and facility referrals. Members of microfinance groups not randomized to receive ICB continued to receive usual clinical care for HIV and other chronic conditions at health facilities within their county. The Harambee study also prospectively followed a nonrandomized comparison group of trial-eligible patients with HIV who were receiving usual facility-based care and not participating in group microfinance.

Group microfinance activities were implemented through the AMPATH-affiliated Group Integrated Savings for Health Empowerment (GISHE) model. These groups are community-based savings and lending associations in which members contribute to a shared fund that can be borrowed by other members for small business investments or emergency needs. Meetings were held regularly in community settings selected by group members. Microfinance groups have been shown to foster social support, trust, and accountability among participants, providing an existing structure for delivering community-based care.^27,28^

### Participants

Microfinance group members were invited to participate in the trial if the following inclusion criteria were met: at least 18 years of age at study baseline, HIV-positive, received HIV-related care through AMPATH after 2010, initiated on ART at least 6 months before study baseline, consistently attended GISHE group meetings within the last 6 months before study baseline and were actively engaged in saving and lending, had an AMPATH Medical Record System (AMRS) ID, and were willing and able to provide written informed consent. Sixty-one microfinance groups (1112 participants) were invited to participate. Following trial exclusions, 57 microfinance groups (855 participants) were enrolled and underwent randomization in a 1:1 ratio to either microfinance groups receiving integrated community-based care (MF + ICB, n = 407 PLHIV) or microfinance groups receiving usual (facility-based) care (MF + UC, n = 448 PLHIV). Microfinance group participants were matched to an additional 300 AMPATH patients who received usual facility-based care and were not engaged in group microfinance (UC). AMPATH provides HIV care at no cost to patients, including ART. Therefore, all participants included in the current study received ART and associated care at no cost.

In the Harambee study, some participants received care at medical facilities that maintained only paper-based records. As part of the current study design to assess ART adherence, a decision was made a priori to limit data collection at these facilities to those with at least 5 enrolled Harambee participants, to reduce the burden of in-person record abstraction. As a result, the analytic sample for this study was reduced (MF + ICB, n = 328 PLHIV and MF + UC, n = 426 PLHIV). In the UC group, 3 patients were lost to follow-up before ART data could be recorded (UC, n = 297 PLHIV).

### Data

We used survey data ascertained for all participants during the first study visit following the study start (month 0) and at the last study visit (month 18). Participants engaged in group microfinance provided an additional round of survey data in study month 9. Survey data assessed the following participant-level covariates: employment status (Demographic & Health Survey, DHS, Wealth Index),^29^ food security (Household Food Insecurity Access Scale),^30,31^ social support (Oslo Social Support Scale),^32^ and internalized HIV stigma (Felt Stigma Questionnaire Among Persons Living with HIV).^33^ We included age, gender, and education status as demographic data.

### Outcome Variables

Our primary outcome of interest was the 18-month change in primary ART adherence through the MPR. The MPR is a common indicator of medication adherence that estimates the proportion of days a patient has ART available over a specified time, based on pharmacy dispensing data.^34^ It reflects consistent medication access and persistence in treatment and is shown to be strongly associated with HIV viral suppression.^5,6^ We calculated MPR as the total number of days’ supply of ART dispensed during an observation period divided by the total number of days in that period (Supplemental Equation 1a). We obtained prescription ART data for MF + ICB through dispensing records during ICB care visits. An in-person review of all electronic and paper-based medical records kept by 21 local facilities serving trial participants in Trans Nzoia and Busia counties was conducted to ascertain ART data for MF + UC. For patients in the UC group, we reviewed the electronic AMRS for ART data. To ensure consistency, all ART data, whether obtained from intervention visits, facility records, or the AMRS, were abstracted using standardized procedures uniformly across study arms.

To assess the 18-month change in MPR, we examined data beginning with each participant's first ART prescription refill following study initiation and constructed 3 time-interval-based measures. The first (baseline) MPR consisted of the measure calculated over 0-6 months for MF + UC and UC groups and over 4-6 months for the MF + ICB group. The second (mid-intervention) and third (late-intervention) MPR were calculated for all groups over months 6-12 and 12-18, respectively. The MPR was capped at 1 to prevent overestimation that could occur from early fills. We reported the MPR as a continuous variable for our analyses. We interpreted the MPR as “optimal” if >0.94, “suboptimal” if 0.80-0.94, and “poor” if <0.80 based on existing literature associating MPR with HIV virologic failure.^35–37^

Our secondary variable of interest was patient-reported ART adherence measures at months 0 and 18, measured by the self-reported 4-day ART adherence ratio assessed via the AIDS Clinical Trials Group (ACTG) Adherence Questionnaire.^38^ The self-reported measure is a continuous variable representing the number of prescribed doses missed during the prior 4 days (Supplemental Equation 2a). While the MPR provides objective data on medication availability, the 4-day ART adherence ratio captures short-term self-reported adherence at the beginning and end of the Harambee study. Including both allowed us to assess complementary dimensions of adherence and evaluate consistency between self-reported and objective measures.

### Statistical Analysis

We compared baseline participant demographic data of relevant covariates using χ2 tests for categorical variables and *t*-tests for continuous variables. The specified significance threshold was a *P*-value <0.05 with 2-sided tests throughout the analyses. To visually assess changes over the study period, we graphed the mean MPR for all groups during the baseline, mid-intervention, and late-intervention periods. To evaluate changes in participant-reported ART adherence, we graphed the change in the mean 4-day ART adherence ratio at months 0 and 18.

We used linear regression models with treatment-by-time interactions and individual- and month-fixed effects to evaluate the impact of ICB care on pharmacy-reported adherence compared to facility-based care with and without microfinance participation (Supplemental Equation 3a). As a secondary analysis, we assessed the impact of the study interventions on the 4-day ART adherence ratio (Supplemental Equation 4a). We clustered standard errors at the level of the microfinance group, where applicable, and the level of the individual in usual care patients to account for potential correlation between individuals within each group. We defined the post-treatment period as starting from month 6 onward, indicated by a binary variable *post time* where *post time* = 1 if the month is ≥6, and 0 otherwise.

### Sensitivity Analysis

We conducted robustness checks to determine the validity of our model's results on our primary analysis (MPR). To improve the precision of our estimates, we included baseline participant-level covariates that previously published literature predicted may be associated with changes in ART adherence. To address the possible impacts of missing data on our primary analysis, we assessed missingness predictors by examining lagged participant-level covariates. Based on this, we implemented inverse probability weighting (IPW) to adjust for the likelihood of having complete ART data. Weights were constructed using observed covariates and applied within a weighted treatment-by-time regression framework to reduce bias from data that may be missing not at random.

All analyses were conducted with R (version 4.3.2, R Project for Statistical Computing).

## Results

### Primary Analysis

A total of 1051 PLHIV were included in the analysis (MF + ICB, n = 328 PLHIV; MF + UC, n = 426 PLHIV; and UC, n = 297 PLHIV). Table 1 presents covariates and baseline demographic data by the 3 groups. The mean age at enrolment was 52.4 years in MF + ICB, 51.6 years in MF + UC, and 52.6 years in the UC group. Most participants were female (74.6% in MF + ICB, 74.2% in MF + UC, and 76.8% in UC) and had at least primary-level education (84.2% in MF + ICB, 69.7% in MF + UC, and 83.8% in UC). Members of microfinance groups (MF + ICB and MF + UC) self-reported significantly higher levels of social support than matched patients receiving usual care without microfinance (UC). Usual care patients had a significantly lower level of reported HIV stigma and unemployment than MF + UC participants. Participants in MF + ICB and MF + UC did not differ significantly in baseline covariates.

**Table 1.** Baseline Demographic Data and Preexposure Covariates for Harambee Study Participants.

	MF + ICB (A) (*N* = 328)	MF + UC (B) (*N* = 426)	UC (C) (*N* = 297)	*P*-values A to B; A to C; B to C
**Age at enrollment (years)**				
Mean (SD)	52.4 (10.7)	51.6 (11.6)	52.6 (11.0)	0.05; 0.715; 0.657
Median [Min, Max]	53.0 [25.0, 90.0]	52.0 [23.0, 89.0]	53.0 [1.00, 78.0]	
**Gender**				
Male	82 (25.4%)	110 (25.7%)	69 (23.3%)	0.96; 0.596; 0.48
Female	241 (74.6%)	316 (74.2%)	228 (76.8%)	
**Education status**				
None	51 (15.8%)	81 (19.0%)	48 (16.2%)	0.070; 0.987; 0.103
Primary schooling and above	272 (84.2%)	297 (69.7%)	249 (83.8%)	
Missing	0 (0%)	48 (11.3%)	0 (0%)	
**Employment**				
Unemployed/Not working	82 (25.4%)	115 (27.0%)	68 (23.9%)	0.163; 0.544; 0.038
Employed/Working	241 (74.6%)	263 (61.7%)	228 (76.8%)	
Missing	0 (0%)	48 (11.2%)	1 (0.3%)	
**Social support**				
Poor or no social support present	133 (41.2%)	174 (40.8%)	161 (54.2%)	0.433; <0.001; 0.08
Social support present	140 (43.3%)	159 (37.3%)	93 (31.3%)	
Missing	50 (15.5%)	93 (21.8%)	43 (14.5%)	
**Internalized HIV stigma**				
Low stigma	284 (87.9%)	340 (79.8%)	249 (83.8%)	0.464; 0.178; 0.025
High stigma	39 (12.1%)	38 (8.9%)	48 (16.2%)	
Missing	0 (0%)	48 (11.3%)	0 (0%)	
**Food security**				
Little to no household hunger present	119 (36.8%)	115 (27.0%)	95 (32.0%)	0.086; 0.236; 0.725
Moderate-to-severe household hunger present	204 (63.2%)	263 (61.7%)	202 (68.0%)	
Missing	0 (0%)	48 (11.3%)	0 (0%)	

*Notes*: Differences between study arms were assessed using χ^2^ tests for categorical variables and *t*-tests for continuous variables.

MF, group microfinance; ICB, integrated community-based care; UC, usual care.

**Figure 1.**
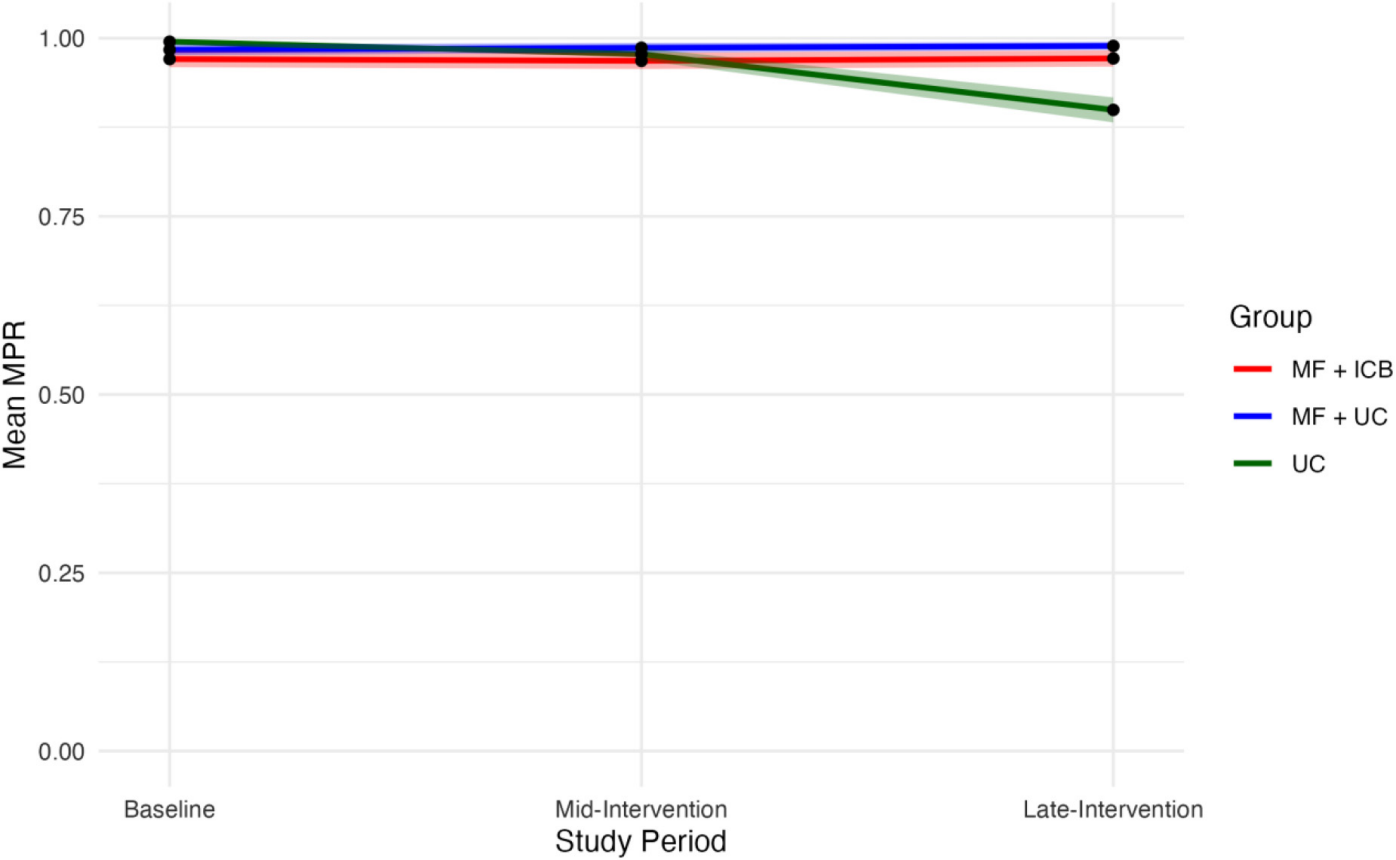
Changes in mean MPR throughout the 18-month study period by group. The 95% confidence intervals are shown as shaded areas. Baseline 4-day ART adherence ratio is measured at month 0. Late-intervention 4-day ART adherence ratio is measured at month 18. MF, group microfinance; ICB, integrated community-based care; MPR, medication possession ratio; UC, usual care.

Figure 1 illustrates changes in the mean MPR over the 18-month study. For the baseline interval, mean MPR measures were similar across groups (MF + ICB = 0.971; 95% CI = 0.959-0.982, MF + UC = 0.989; 95% CI = 0.976-0.991, and UC = 0.995; 95% CI = 0.989-1). Compared to the baseline interval, the MPR increased during the mid-intervention period for all groups (MF + ICB = 0.968; 95% CI = 0.960-0.980, MF + UC = 0.986; 95% CI = 0.980-0.993, and UC = 0.978; 95% CI = 0.969-0.986). During the late-intervention period, the UC group's mean MPR declined noticeably (UC = 0.899; 95% CI = 0.881-0.917) compared with participants engaged in microfinance (MF + ICB = 0.971; 95% CI = 0.960-0.983 and MF + UC = 0.989; 95% CI = 0.983-0.995).

In our primary analysis using a treatment-by-time model (Table 2), participants in both intervention arms experienced significantly greater improvements in MPR over time compared to the UC group. Specifically, MPR increased by 0.048 in the MF + ICB group (*P* < 0.001) and by 0.057 in the MF + UC group (*P* < 0.001), relative to the UC group. Post-treatment time (>6 months) and month-fixed effects were statistically significant and associated with decreased MPR in these models. When comparing MF + ICB to MF + UC, there was a slight nonsignificant decrease in the MPR (MF + ICB β = −0.008; *P* = 0.459). Post-treatment time (>6 months) and the month-fixed effect were not statistically different between the two groups engaged in microfinance.

**Table 2.** Effects of Group Microfinance With Integrated Community Care or with Usual Facility Care, Compared to Usual Care Without Microfinance on ART Medication Possession Ratio: Treatment-by-Time Model.

Model term	Estimate	Std error	T-value	*P*-value
Post time	−0.047	0.006	−7.343	**<0**.**001**
MF + ICB × Post time	0.048	0.008	5.678	**<0**.**001**
MF + UC × Post time	0.057	0.007	7.191	**<0**.**001**
MF + ICB Compared to MF + UC × Post time	−0.008	0.012	−0.741	0.459
Month-fixed effects	−0.022	0.004	−5.969	**<0**.**001**

*Notes*: Interaction terms represent the treatment-by-time estimators for patient-level ART MPR. This model includes individual-level fixed effects and month-fixed effects. Robust estimators and standard errors are clustered at the study-group level. Post time is a dummy variable = 1 if the month ≥ 6, and 0 otherwise, indicating the post-treatment period. Interaction terms involving post time and treatment group variables represent the difference in treatment effects between the post-treatment and pre-treatment periods.

ART, antiretroviral therapy; MF, group microfinance; ICB, integrated community-based care; MPR, medication possession ratio; UC, usual care.

### Secondary Analysis

**Figure 2.**
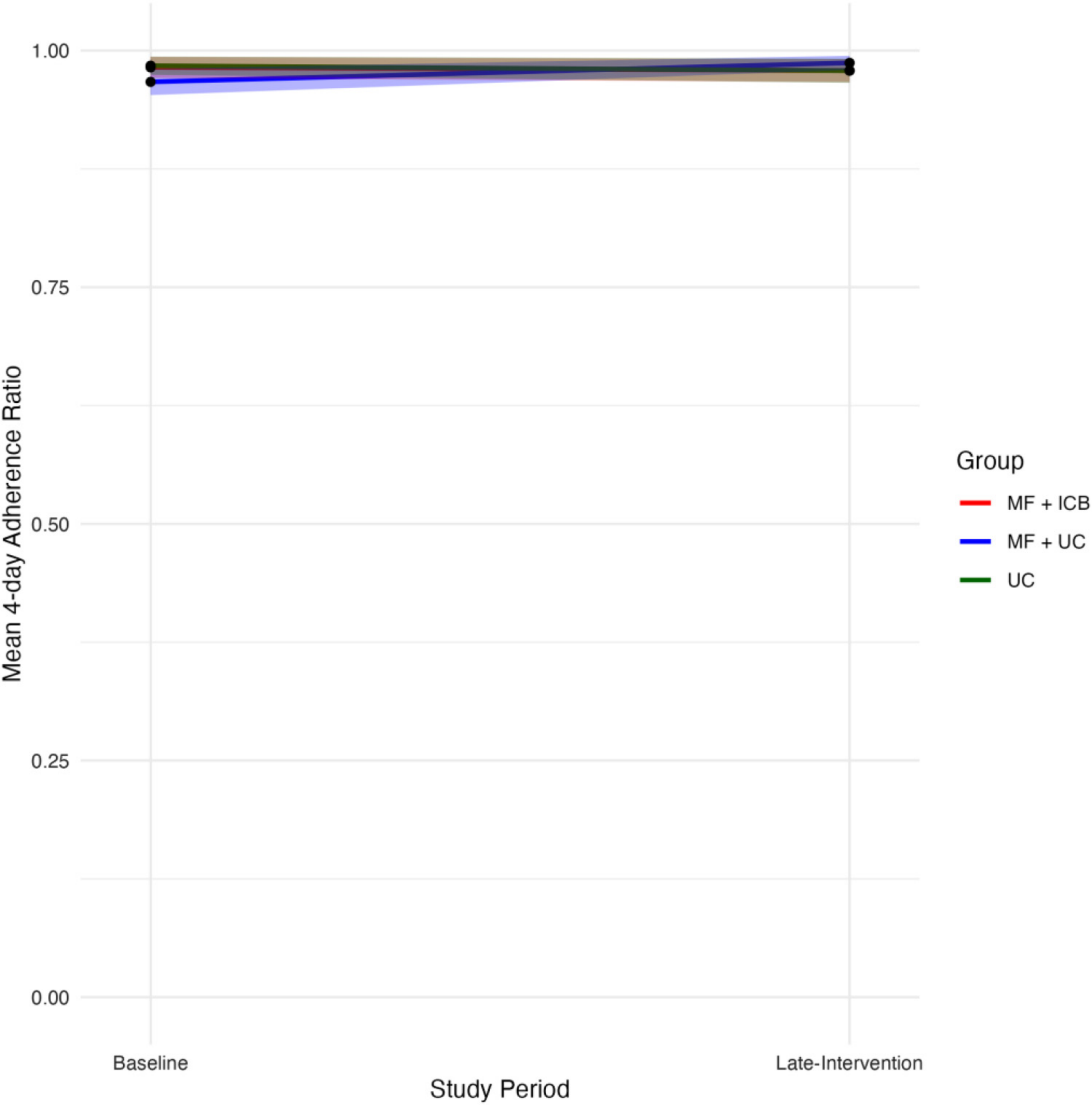
Changes in mean 4-day ART adherence ratio based on the 4-day ART adherence ratio assessed via the AIDS Clinical Trials Group (ACTG) Adherence Questionnaire, throughout the 18-month study period by group. The 95% confidence intervals are shown as shaded areas. Baseline 4-day ART adherence ratio is measured at month 0. Late-Intervention 4-day ART adherence ratio is measured at month 18. ART, antiretroviral therapy; MF, group microfinance; ICB, integrated community-based care; MPR, medication possession ratio; UC, usual care.

Figure 2 shows the mean 4-day ART adherence ratio measures for all 3 groups at months 0 and 18. At baseline, ratios were not visually different (MF + ICB = 0.982; 95% CI = 0.971-0.993, MF + UC = 0.967; 95% CI = 0.953-0.981, and UC = 0.984; 95% CI = 0.974-0.994). At month 18, the mean 4-day ART adherence ratio slightly decreased for the MF + ICB and the UC group, and slightly increased for the MF + UC group (MF + ICB = 0.978; 95% CI = 0.966-0.991, MF + UC = 0.987; 95% CI = 0.980-0.994, and UC = 0.978; 95% CI = 0.974-0.994). Table 3 shows the impact of ICB care compared to usual care with and without group microfinance on patient-reported ART adherence. Only the MF + UC group saw a significantly increased 4-day ART adherence ratio (MF + UC β = 0.021; *P* = 0.05). All other effects were nonsignificant.

**Table 3.** Effects of Group Microfinance With Integrated Community Care or With Usual Facility Care, Compared to Usual Care Without Microfinance on Self-Reported 4-Day ART Adherence Ratio: Treatment-by-Time Model.

Model term	Estimate	Std error	*T*-value	*P*-value
Post time	−0.007	0.009	−0.721	0.471
MF + ICB × Post time	0.001	0.001	0.100	0.920
MF + UC × Post time	0.021	0.010	1.923	**0**.**05**
MF + ICB Compared to MF + UC × Post time	−0.020	0.016	1.27	0.203
Month-fixed effects	0.002	0.008	1.213	0.226

*Notes*: Interaction terms represent the treatment-by-time estimators for patient-level 4-day ART adherence ratio. This model includes individual-level fixed effects and month-fixed effects. Robust estimators and standard errors are clustered at the study group level. Post time is a dummy variable = 1 if the month ≥ 6, and 0 otherwise, indicating the post-treatment period. Interaction terms involving post time and treatment group variables represent the difference in treatment effects between the post-treatment and pre-treatment periods.

ART, antiretroviral therapy; MF, group microfinance; ICB, integrated community-based care; UC, usual care.

### Sensitivity Analysis

We included *high/moderate social support present*, *presence of household hunger*, education status (*primary school and above*), *unemployment or not working*, and *high internal HIV stigma* in the primary analysis for robustness checks. Including participant-level covariates did not influence the significance of the effects observed on pharmacy-reported ART adherence, except for month-fixed effects, which were no longer significant (Supplemental Table 1a). We observed slight increases in effect size on the MPR compared to our primary analysis when including participant-level covariates for both groups engaged in microfinance (MF + ICB β = 0.081; *P* < 0.001 and MF + UC β = 0.096; *P* < 0.001).

During the late-intervention period, 68 participants did not have ART fill data. We observed higher missing ART refill data levels in the MF + ICB group (MF + ICB = 36; 52.9%, MF + UC = 13; 19.1%, and UC = 19; 27.9%). We evaluated preexposure participant-level covariates to determine if participants with missing data differed from those without missingness. We found individuals with missing MPR data in the late-intervention period were more likely to report high levels of internalized HIV stigma compared to their counterparts without missing data (non-missing = 115; 10.6%, missing = 15; 22.1%, *P* = 0.009). We assumed that missingness within this analysis was not at random due to the significant difference in the HIV-stigma covariate. We utilized IPW to assign greater weights to participants with high levels of reported internalized HIV stigma and lower weights to those with low reported levels. We applied these weights within a weighted treatment-by-time regression and found the results consistent with our primary analysis (MF + ICB β = 0.088; *P* < 0.001, MF + UC β = 0.099; *P* < 0.001).

## Discussion

We leveraged panel data from a cluster randomized trial among 57 microfinance groups and 1051 adults living with HIV in western Kenya to assess the impact of combining integrated community-based care with group microfinance on objectively measured and self-reported ART adherence. We found that ICB care with group microfinance and group microfinance with usual care (MF + ICB and MF + UC) were associated with significant increases in MPR compared to usual care (UC) without microfinance. In this study, the mean MPR was relatively high across all participants during the 18-month study period, except for those in the UC group, where the MPR decreased to “suboptimal” in the late-intervention period. The 4-day ART adherence ratio increased significantly in the MF + UC group.

Despite declines in the primary ART adherence in the UC group, patient-reported adherence remained elevated throughout the study period in all 3 groups. In the UC group, when the mean MPR was suboptimal (0.899), the mean 4-day ART adherence ratio was 0.978, indicating, on average, participants adhered to their ART within the previous 4 days, 97.8% of the time. Patient-reported adherence through the 4-day ART adherence ratio from the AACTG follow-up questionnaire is a commonly used measure of adherence in resource-limited settings. However, it can suffer from recall bias, as indicated in our study results.^39–41^

Though we observed relatively high primary ART adherence throughout the study, the mean MPR of the UC group fell below 0.94 to 0.899, resulting in “suboptimal adherence.” HIV viral suppression relies upon a high level of ART adherence to prevent viral replication and subsequent mutation. Even modest improvements in adherence, as seen in the MF groups, can be clinically meaningful. Our results are consistent with the main Harambee study findings, which demonstrated significantly higher levels of HIV viral suppression at 18 months among participants in both MF + ICB and MF + UC groups compared to the UC group^42^ Kenya's recent introduction of a dolutegravir-containing single-pill regimen has increased access for millions of Kenyans, but HIV drug resistance is increasing, underscoring the need for targeted efforts to sustain adherence and increase viral suppression.^43^

In SSA, community-based care is increasingly diversifying HIV management to increase care delivery and retention.^15,16^ Increased care retention coupled with continual access to ART and clinical monitoring can increase viral suppression and improve HIV-related outcomes.^12^ Community-based care relies on sustainability to deliver care in settings outside the clinic, and budgetary constraints and lack of personnel can restrict the startup or longevity of these programs.^44,45^ Here, we found that participation in group microfinance significantly increased levels of ART adherence compared to usual care alone. We found no significant increases in either ART measure when comparing ICB care to group microfinance with usual care. In areas that do not have access to community-based programs, group microfinance may serve to improve ART adherence.

Prior studies indicate that engagement in microfinance groups or other economic strengthening interventions within low-and middle-income countries is associated with increased ART adherence.^46^ It is theorized that social support through the “group effect,” increased economic support, and decreased vulnerability in microfinance group participants may aid in increasing ART adherence.^46,47^ In the Harambee study, all microfinance groups were cluster randomized and composed mainly of PLHIV. We observed that participants engaged in group microfinance had significantly higher levels of social support, potentially attributed to their involvement in microfinance. Education and training sessions within microfinance group meetings, specifically those that focus on ART and HIV, may also be a pathway through which group microfinance improves medication adherence.^47,48^ Despite AMPATH relieving the direct economic burden associated with ART and HIV-related care, HIV patients are still susceptible to increased indirect costs.^49^ Group microfinance may help to alleviate the economic burden of indirect costs these patients experience. In the case of community-based care, integrating these services in a strong social setting may strengthen care delivery. Microfinance group meetings may offer a more supportive environment for community-based care integration, where individuals sharing similar disease states can receive tailored support and assistance without external stigma.^27,45,50,51^

### Limitations

Our study has several limitations. First, the MPR as an adherence measure cannot fully capture all behaviors that comprise adherence, such as medication ingestion, dosing intervals, medication interactions, or dietary interactions that, in the case of HIV, can influence viral suppression. Still, multiple studies in SSA on ART adherence have found that an optimal MPR (>0.94) is associated with improved clinical outcomes such as increased CD4 + lymphocyte count and viral suppression.^36,37^ Although the observed but statistically significant MPR differences were modest in magnitude, small improvements in adherence may yield meaningful clinical and public health benefits. Our results were supported by the main findings from the Harambee study, which demonstrated significantly higher HIV viral suppression at 18 months among participants in both the MF + ICB and MF + UC groups compared to matched controls.^42^ Second, microfinance groups receiving ICB care experienced a period of ART alignment from months 0-3, where participants with existing ART received smaller quantities of medication to prevent over-dispensing and stockpiling of medication. Because of this, we could not ascertain a reliable MPR for these 3 months and had to shorten the baseline MPR in the MF + ICB group to 4-6 months. Even then, MF + ICB participants were under the greatest level of clinical oversight, with ART delivered directly to the community. If anything, restricting the MF + ICB group's baseline may bias our observed ICB effect to the null. Third, there is the variation in data collection used to calculate the MPR. Specifically, some MPR data were extracted from paper-based medical records, while others were obtained from the electronic AMRS. These differences could have introduced inconsistencies in the accuracy of the data. To mitigate this, we applied a standardized approach to data extraction across data sources and conducted quality checks where feasible. Nonetheless, residual discrepancies between collection methods may have influenced the results and should be considered when interpreting our findings. Fourth, although the Harambee study conducted a power calculation, this secondary analysis did not perform a formal power calculation for the outcomes assessed.^19^ This should be considered when interpreting the findings. We also observed nontrivial rates of missing data in our primary analysis. Participants with missing data had significantly higher levels of internalized HIV stigma than non-missing participants, which may indicate a missing pattern not at random. To address this concern, we performed a weighted regression analysis using IPW, which indicated no significant deviation from the outcomes observed in our primary model. Lastly, our study population was comprised mainly of participants engaged in HIV care with AMPATH for a significant time prior to this study, so the results we find here may not be generalizable to other patient groups. Future work should focus on the impacts of ICB care or participation in group microfinance in other study populations less engaged in HIV care, and directly test specific mechanisms mediating the relationship from microfinance participation to ART adherence.

## Conclusion

Group microfinance participation may increase ART adherence through increased social support and education. Integrating community-based care into a microfinance setting could notably improve HIV-care delivery and adherence to ART among PLHIV in resource-limited areas with high levels of stigma. Further research should focus on understanding the effects of these interventions on patient populations less engaged in HIV care.

## Supplemental Material

sj-docx-1-jia-10.1177_23259582251414566 - Supplemental material for Effects of Integrated Community-Based Care and Group Microfinance on Antiretroviral Therapy Adherence Among Adults Living With HIV in Western Kenya Supplemental material, sj-docx-1-jia-10.1177_23259582251414566 for Effects of Integrated Community-Based Care and Group Microfinance on Antiretroviral Therapy Adherence Among Adults Living With HIV in Western Kenya by Emily T. O’Neill, Juddy Wachira, Joshua Juma, Ben Mosong, Catherine Kafu, Marta Wilson-Barthes, Sonak D. Pastakia, Dan N. Tran, Becky L. Genberg and Omar Galárraga in Journal of the International Association of Providers of AIDS Care (JIAPAC)

sj-docx-2-jia-10.1177_23259582251414566 - Supplemental material for Effects of Integrated Community-Based Care and Group Microfinance on Antiretroviral Therapy Adherence Among Adults Living With HIV in Western Kenya Supplemental material, sj-docx-2-jia-10.1177_23259582251414566 for Effects of Integrated Community-Based Care and Group Microfinance on Antiretroviral Therapy Adherence Among Adults Living With HIV in Western Kenya by Emily T. O’Neill, Juddy Wachira, Joshua Juma, Ben Mosong, Catherine Kafu, Marta Wilson-Barthes, Sonak D. Pastakia, Dan N. Tran, Becky L. Genberg and Omar Galárraga in Journal of the International Association of Providers of AIDS Care (JIAPAC)
